# Dysregulated cytokine profile associated with biochemical premature ovarian insufficiency

**DOI:** 10.1111/aji.13292

**Published:** 2020-06-26

**Authors:** Peihao Liu, Xiruo Zhang, Jingmei Hu, Linlin Cui, Shidou Zhao, Xue Jiao, Yingying Qin

**Affiliations:** ^1^ Center for Reproductive Medicine Cheeloo College of Medicine Shandong University Jinan China; ^2^ National Research Center for Assisted Reproductive Technology and Reproductive Genetics Shandong University Jinan China; ^3^ Key laboratory of Reproductive Endocrinology of Ministry of Education Shandong University Jinan China; ^4^ Shandong Provincial Clinical Medicine Research Center for Reproductive Health Shandong University Jinan China

**Keywords:** chemokine, cytokine, growth factor, premature ovarian insufficiency

## Abstract

**Problem:**

Premature ovarian insufficiency (POI) imposes great challenge on female reproduction. Whether immune disturbance in ovarian environment was implicated in POI remains unclear. We aimed to characterize the cytokine profile in follicular fluid (FF) and paired serum in patients with biochemical POI (bPOI).

**Method of study:**

Multiplex immunoassay containing 45 cytokines was performed for individual FF and paired serum samples from 35 bPOI patients and 37 matched controls. Cytokine profiles were compared between the two groups and cytokines correlated to ovarian reserve, and the rates of day‐3 good‐quality embryos were further analyzed.

**Results:**

In FF, significantly elevated level of chemokines MIP‐1α (*P* = .043), CXCL8 (*P* = .024), IP‐10 (*P* = .041), and eotaxin‐1 (*P* = .015) as well as growth factors VEGF‐D (*P* = .047), BDNF (*P* = .043), LIF (*P* = .002), and bFGF (*P* = .046) was found in bPOI patients compared to controls. Yet RANTES manifested an opposite trend with reduced levels among bPOI patients (*P* = .006). All these chemokines and growth factors in FF were significantly correlated with ovarian reserve (*P* < .05). In paired serum, cytokine signature was not likely accordant with that in FF between two groups, except for increased IP‐10 (*P* = .032) in bPOI patients and its significant correlation to FSH and AFC (*P* < .05). Among all differentially expressed cytokines, RANTES in FF was correlated with the rate of day‐3 good‐quality embryos (*P* = .035).

**Conclusion:**

Altered cytokine profile characterized by increased chemokines and growth factors was associated with early stage of POI, which may fuel the progression of the disease or even play a crucial role in the development of ovarian insufficiency.

## 1 INTRODUCTION

Premature ovarian insufficiency (POI), also termed as primary ovarian insufficiency (POI), refers to the decline or cessation of ovarian function prior to age 40. It encompasses the spectrum of ovarian dysfunction from decreased fecundity (occult POI), and elevated follicle‐stimulating hormone (FSH) (biochemical POI) to amenorrhea (overt POI).^1^, ^2^, ^3^ It is estimated that POI affects 1%‐5% of women before the age of 40.^4^ As a major cause of infertility, POI represents one of the most challenging issues in female reproduction. Upon confirmed diagnosis, available ovarian function seldom exists. Additionally, there is no effective strategy to improve or rescue ovarian function and fertility currently. Therefore, it is of great importance to identify risk factors of ovarian insufficiency at earlier stage. However, studies were mainly conducted in women with fully developed overt POI and failed to determine biomarkers or indicators that could accurately predict the status or progress of POI.

Premature ovarian insufficiency is pathophysiologically complicated and etiologically heterogeneous. The known causes include genetics, autoimmunity, infectious, and iatrogenic. Still, most cases remain elusive.^5^ The autoimmune disturbance explains 5%‐30% of POI cases, and it has been attracting increasingly clinical and scientific concerns.^6^ How autoimmunity induces ovarian senescence has not been fully established. No specific, reliable, and non‐invasive diagnostic tests for autoimmune POI exist currently.^7^ Especially, in contrast to accumulated evidence of peripheral autoimmune dysregulation reported, local ovarian autoimmunity was seldom explored in patients with POI.

Follicular microenvironment is critical for folliculogenesis and acquisition of oocyte competence.^8^, ^9^ Cytokines in follicular fluid are increasingly recognized as potential modulators of ovarian function either by autocrine or paracrine mechanisms. A cascade of cytokines, chemokines, and growth factors could mediate the interaction among lympho‐hematopoietic cells, somatic cells and oocytes, and involve in follicular growth, steroidogenesis, activation of leukocytes necessary for ovulation, and tissue remodeling during ovulation, luteinization, and luteolysis.^10^, ^11^ Inflammation negatively affects the follicular microenvironment and thereby reduces oocyte quality and quantity.^12^ Increased levels of pro‐inflammatory cytokines, such as interleukin IL‐6 and CXCL8, in follicular fluid were associated with reduced oocyte quality.^8^, ^13^ Moreover, oocyte quality was decreased in pathologies associated with local follicular inflammation, including aging and polycystic ovary syndrome (PCOS).^14^ However, it is currently unclear whether local inflammation in follicular environment induced dysregulated cytokine milieu is associated with the development of POI.

In this study, given bPOI is easier to diagnose and represents the early stage of ovarian insufficiency, the follicular fluid and paired serum from patients with bPOI were collected. Cytokine profiles were characterized in follicular fluid and paired serum in patients with biochemical POI synchronously to determine specific cytokine signature involved, which would be of great value to explain the decline of ovarian function and provide new avenues for early intervention.

## 2 MATERIALS AND METHODS

### 2.1 Participants

The study recruited subjects undergoing their first in vitro fertilization (IVF)/intracytoplasmic sperm injection (ICSI) cycle at the Reproductive Hospital Affiliated to Shandong University from January 2016 to June 2017. The inclusion criteria of biochemical POI (bPOI) included age <38 years, serum FSH > 10 IU/L, and AMH ≤ 1.1 ng/mL. Those women aged below 38 years with normal ovarian reserve diagnosed by FSH < 10 IU/L and AMH > 1.1 ng/mL, who received IVF/ICSI treatment because of tubal or male factors, were enrolled as controls. And the controls were patients who underwent oocyte retrieval on the same day as the bPOI participants with matched age and BMI. Participants with PCOS, recurrent spontaneous abortion, endometriosis, chromosomal abnormalities, history of ovarian surgery, chemo‐/radio‐therapy, or autoimmune disorders were excluded. The study was approved by the Ethical Committee of Reproductive Medicine of Shandong University. Written informed consents were obtained from all participants.

### 2.2 Endocrine hormones and ultrasonography

Endocrine hormones—including follicle‐stimulating hormone (FSH), luteinizing hormone (LH), prolactin (PRL), estradiol (E2), thyroid‐stimulating hormone (TSH), and testosterone—were detected on day 2‐4 of the menstrual cycle through chemiluminescence immunoassay (Roche Diagnostics). Anti‐Müllerian hormone (AMH) was measured with ultra‐sensitive enzyme‐linked immunosorbent assay (Kangrun Biotech). The intra‐ and inter‐assay coefficients of variation were <10% and <15%, respectively. Antral follicle count (AFC) was recorded as the number of follicles (2‐10 mm in diameter) through transvaginal ultrasonography in the early follicular phase.

### 2.3 Serum and follicular fluid collection

Peripheral blood was sampled on day 3 of menstrual cycle, and serum was isolated and stored at −80°C until assayed. Paired follicular fluid from dominant follicles containing oocytes (diameter ≥ 1.4 cm) was collected during oocyte retrieval, separated, and stored at −80°C until used. Only follicular fluid without blood or flushing solution was collected.

### 2.4 Cytokine profiling

Individual follicular fluid and serum samples were subjected to cytokine profile measurement. A total of 45 cytokines, chemokines, or growth factors were measured using magnetic beads multiplex immunoassay from Luminex™ with the ProcartaPlex Human Cytokine Chemokine Growth Factor (Thermo Fisher Scientific). These included brain‐derived neurotrophic factor (BDNF), eotaxin‐1/CCL11, basic fibroblast growth factor (bFGF), epidermal growth factor (EGF), granulocyte‐macrophage colony‐stimulating factor (GM‐CSF), growth related oncogene‐α(GRO‐α)/CXCL1, hepatocyte growth factor (HGF), nerve growth factor‐β (bNGF), leukemia inhibitory factor (LIF), interferon‐α (IFN‐α), interferon‐γ (IFN‐γ), interleukin‐1β (IL‐1β), IL‐1α, IL‐1RA, IL‐2, IL‐4, IL‐5, IL‐6, IL‐7, IL‐8/CXCL8, IL‐9, IL‐10, IL‐12p70, IL‐13, IL‐15, IL‐17A, IL‐18, IL‐21, IL‐22, IL‐23, IL‐27, IL‐31, interferon inducible protein‐10 (IP‐10)/CXCL10, monocyte chemotactic protein‐1 (MCP‐1)/CCL2, macrophage inflammatory protein‐1α (MIP‐1α)/CCL3, macrophage inflammatory protein‐1 β (MIP‐1β)/CCL4, RANTES/CCL5, stromal cell derived factor (SDF‐1α)/CXCL12, tumor necrosis factor‐α (TNF‐α), tumor necrosis factor‐β (TNF‐β), platelet‐derived growth factor‐BB (PDGF‐BB), placenta growth factor‐1 (PIGF‐1), stem cell factor (SCF), vascular endothelial growth factor‐A (VEGF‐A), and vascular endothelial growth factor‐D (VEGF‐D). Briefly, after coating with magnetic beads, microplates were incubated with 25 μL sera or FF for 60 min at room temperature and then washed twice with wash solution. Detection antibody mixture (25 μL) were added and incubated for 30 min. After washing, SAPE solution (50 μL) was applied for 30 min. Then, reading buffer (120 μL) was added to the plates after washing for 5 min and run on a Luminex 200 system (Luminex). The concentration was calculated by plotting the expected concentration of the standards against the MFI generated by each standard. The samples were run in duplicates.

### 2.5 Statistical analyses

Baseline characteristics and cytokine levels were expressed as median with interquartile ranges. Kolmogorov‐Smirnov test was used to evaluate the normality of data distribution. *t* test was used for original data or Napierian logarithm transformed data that were normally distributed, and Mann‐Whitney *U* test was used for the other data to compare between groups. Putting participants from the two groups together, Spearman's correlation was used to estimate the association between cytokines in follicular fluid and the biomarkers of ovarian reserve (FSH, AMH and AFC) as well as between the follicular and serum. According to the Society for Assisted Reproductive Technology (SART) grading system, day‐3 good‐quality embryos were defined as embryos with six or more blastomere and with fragmentation degree ＜25%. The rate of day‐3 good‐quality embryo was defined as number of day‐3 good‐quality embryos divided by the number of 2 pronuclear (2PN) zygotes.^15^ Spearman's correlations between follicular fluid and serum cytokines from all participants were also evaluated. *P* ＜ .05 was considered to be statistically significant. All statistical analyses were performed with SPSS (SPSS Inc, version 21.0).

## 3 RESULTS

In this study, 35 women with bPOI and 37 controls with normal ovarian reserve were enrolled. There were no significant differences regards to age and body mass index (BMI) between the two groups (*P* > .05). Patients with bPOI presented with significantly higher FSH and decreased AMH level and AFC as expected (Table 1).

**TABLE 1:** Demographics and baseline characteristics of the participants

Characteristic	bPOI (n = 35)	CON (n = 37)	*P* value
Age (y)	32.00 (28.00‐34.00)	29.00 (27.00‐31.00)	.059
BMI (kg/m^2^)	21.76 (19.65‐26.67)	22.15 (19.83‐23.88)	.379
AFC	7 (6‐10)	14 (10‐16)	<.001*
FSH (IU/L)	13.75 (12.36‐17.60)	6.35 (5.47‐7.44)	<.001*
LH (IU/L)	5.64 (4.60‐7.60)	5.63 (4.09‐7.15)	.927
Estradiol (pg/mL)	30.10 (22.40‐37.25)	33.20 (26.65‐48.85)	.103
AMH (ng/mL)	0.58 (0.42‐0.92)	2.56 (1.78‐5.02)	＜.001*
Testosterone (ng/mL)	15.42 (10.33‐25.36)	18.84 (12.97‐26.67)	.173
TSH (mIU/mL)	2.47 (1.80‐2.88)	1.97 (1.67‐3.05)	.514
PRL (ng/mL)	13.91 (10.00‐16.65)	15.38 (10.74‐21.85)	.095

* indicates statistical significances of *P* < .05.

Abbreviations: AFC, antral follicle count; AMH, Anti‐Müllerian hormone; BMI, body mass index； FSH, follicle‐stimulating hormone; LH, luteinizing hormone; PRL, prolactin; TSH, thyroid‐stimulating hormone.

**FIGURE 1:**
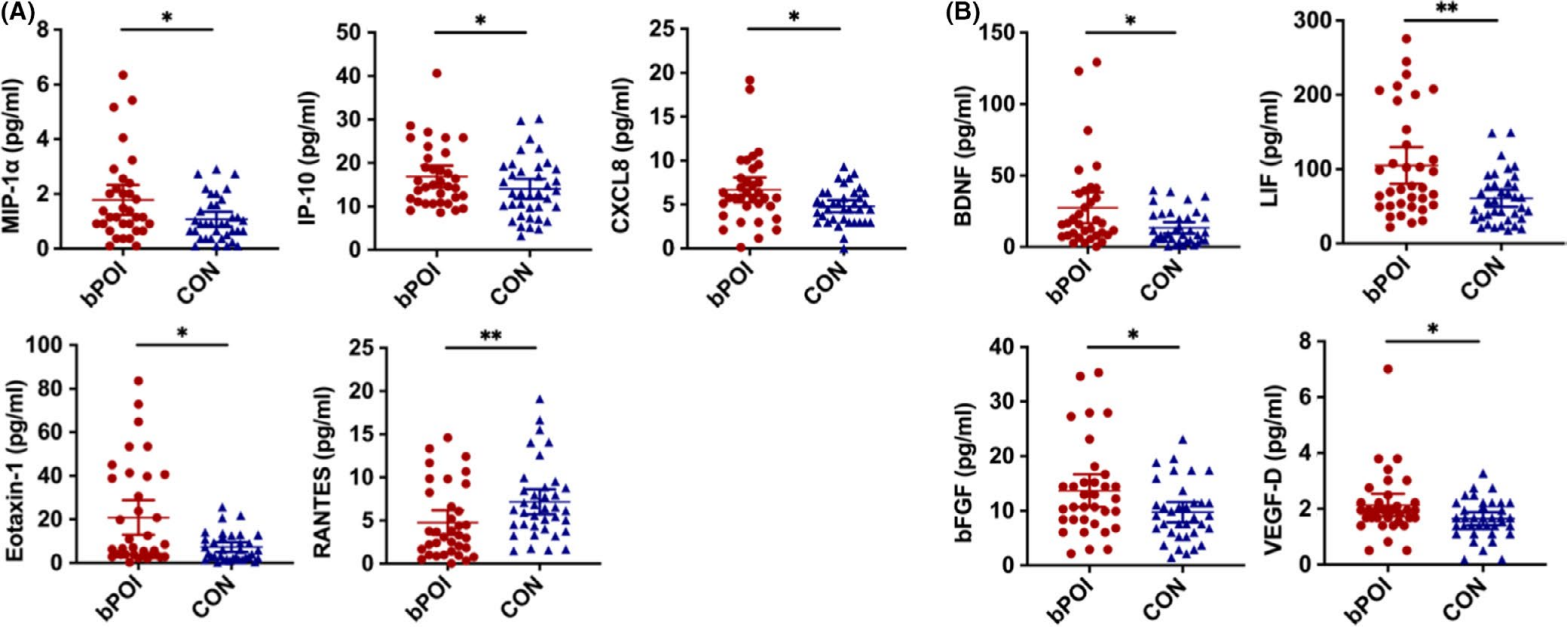
Chemokines and growth factors with significant difference in follicular fluid between patients with bPOI and control women. BDNF, brain‐derived neurotrophic factor; bFGF, basic fibroblast growth factor; IP‐10, interferon inducible protein‐10; LIF, Leukemia inhibitory factor; MIP‐1α, macrophage inflammatory protein‐1α; VEGF‐D, vascular endothelial growth factor‐D

Cytokine profile in FF and paired serum was characterized and compared between patients with bPOI and control women. Interestingly, patients with bPOI had notably distinct cytokine signature in FF, especially for chemokines and growth factors (Table S1). Significantly elevated level of MIP‐1α (*P* = .043), CXCL8 (*P* = .024), IP‐10 (*P* = .041), and eotaxin‐1 (*P* = .015), as well as BDNF (*P* = .043), LIF (*P* = .002), VEGF‐D (*P* = .047), and bFGF (*P* = .046) was found in bPOI patients compared to controls. Yet RANTES manifested an opposite trend with reduced level among bPOI patients (*P* = .006) (Table 2, Figure 1). Additionally, all chemokines and growth factors in FF mentioned above were significantly correlated with at least one biomarker of ovarian reserve. Among these, MIP‐1α, eotaxin‐1, RANTES, BDNF, LIF, bFGF, and VEGF‐D had significant correlation with FSH, AMH, and AFC simultaneously (*P* < .05) (Table 3, and Table S3). Unexpectedly, the level of most interleukin cytokines was very low and showed no difference between two groups except for a higher IL‐27 (*P* = .020) in patients with bPOI (Table S1).

**TABLE 2:** Cytokine profile with statistical significance in serum and follicular fluid between patients with bPOI and control women

Cytokine (pg/mL)	bPOI (n = 35)	CON (n = 37)	*P* value
Follicular fluid			
MIP‐1α	1.15 (0.78‐2.29)	0.91 (0.41‐1.60)	.043
CXCL8	5.76 (4.53‐8.30)	4.46 (2.97‐6.37)	.024
IP‐10	14.78 (11.01‐21.39)	13.09 (8.81‐18.89)	.041
Eotaxin‐1	7.07 (3.62‐39.67)	5.12 (2.18‐12.40)	.015
RANTES	3.31 (1.40‐8.24)	6.26 (4.22‐8.84)	.006
BDNF	16.25 (7.98‐37.93)	8.79 (4.56‐21.91)	.043
LIF	74.77 (50.53‐153.09)	56.85 (33.42‐80.41)	.002
VEGF‐D	1.95 (1.64‐2.29)	1.68 (1.25‐2.22)	.047
bFGF	11.45 (8.20‐15.56)	9.16 (6.05‐11.45)	.046
IL‐27	10.02 (4.29‐16.26)	10.02 (4.29‐10.2)	.020
Serum			
IP‐10	245.97 (188.38‐352.99)	210.95 (157.06‐262.35)	.032
IL‐7	5.12 (2.93‐6.96)	3.75 (2.34‐5.12)	.027
IL‐17A	8.36 (5.35‐11.34)	5.35 (5.35‐7.60)	.017
IL‐1α	0.46 (0.28‐0.62)	0.66 (0.35‐0.89)	.044
IL‐21	23.54 (9.75‐47.05)	42.70 (17.42‐68.97)	.042

Abbreviations: BDNF, brain‐derived neurotrophic factor; bFGF, basic fibroblast groth factor; IL, interleukin; IP, interferon inducible protein; LIF, Leukemia inhibitory factor; MIP, macrophage inflammatory protein; VEGF, vascular endothelial growth factor.

**TABLE 3:** Correlation between follicular fluid cytokines and biomarkers of ovarian reserve

Variables (pg/mL)	FSH (IU/L)		AMH (ng/mL)		AFC	
	Spearman's rho	*P* value	Spearman's rho	*P* value	Spearman's rho	*P* value
MIP‐1α	0.302	.012*	−0.353	.004*	−0.278	.021*
CXCL8	0.202	.090	−0.305	.011*	−0.297	.012*
IP‐10	0.254	.033*	−0.118	.337	−0.211	.077
Eotaxin‐1	0.334	.004*	−0.392	.001*	−0.323	.006*
RANTES	−0.324	.005*	0.349	.003*	0.288	.014*
BDNF	0.325	.006*	−0.323	.008*	−0.342	.004*
LIF	0.367	.002*	−0.325	.007*	−0.309	.008*
bFGF	0.253	.036*	−0.320	.009*	−0.292	.015*
VEGF‐D	0.305	.010*	−0.258	.033*	−0.344	.003*

* indicates statistical significances of *P* < .05.

Abbreviations: BDNF, brain‐derived neurotrophic factor; bFGF, basic fibroblast growth factor; IP, interferon inducible protein; LIF, Leukemia inhibitory factor; MIP, macrophage inflammatory protein; VEGF, vascular endothelial growth factor.

In paired serum, IP‐10 (*P* = .032), IL‐7 (*P* = .027), and IL‐17A (*P* = .017) showed significantly higher levels but IL‐1α (*P* = .044) and IL‐21 (*P* = .042) were decreased in patients with bPOI compared with controls. Cytokine signature in serum was not likely accordant with that in paired FF between two groups, except for increased IP‐10 in patients with bPOI (Table 2 and Table S2). Serum IP‐10 was also found significantly associated with both FSH and AFC (*P* < .05) (Table S4).

To elucidate the association of different follicular and serum cytokines with oocyte quality and embryo quality, the correlation with the rate of day‐3 good‐quality embryos was further analyzed. Among all the differentially expressed cytokines, RANTES in follicular fluid turned out to be correlated with rate of day‐3 good‐quality embryos rate (*P* = .035) (Table 4).

**TABLE 4:** Correlation between cytokines and the rate of day‐3 good‐quality embryos

Variables (pg/mL)	Rate of day 3 good‐quality embryos (%)	
	Spearman's rho	*P* value
Serum		
PIGF‐1	0.258	.034*
GRO‐α	0.308	.010*
Follicular fluid		
RANTES	0.254	.035*
IL‐22	0.252	.037*
GRO‐α	−0.287	.017*
PDGF‐BB	0.246	.041*

* indicates statistical significances of *P* < .05.

Abbreviations: GRO, growth related oncogene; IL, interleukin; PDGF, platelet‐derived growth factor; PIGF, placenta growth factor.

## 4 DISCUSSION

An altered cytokine milieu, especially the distinct profile of chemokines and growth factors in follicular fluid, was identified in the early stage of POI. Significantly elevated level of MIP‐1α, CXCL8, IP‐10, and eotaxin‐1 as well as VEGF‐D, BDNF, LIF, and bFGF were found in bPOI patients compared to controls. RANTES was found decreased in bPOI patients and positively correlated with the rate of day‐3 good‐quality embryos. All these chemokines and growth factors in FF were significantly correlated with ovarian reserve. In paired serum, cytokine signature was not likely accordant with that in FF between two groups, except for increased IP‐10 in bPOI patients and its significant correlation to ovarian reserve.

Ovarian autoimmunity has long been implicated in the pathogenesis of POI. Identification of autoimmune disturbance may enable early interventions possible before ovarian failure occurs. Systemic pro‐inflammatory conditions alter ovarian homeostasis and detrimentally affect follicular dynamics, thus resulting in POI.^12^ Currently, it is unknown whether follicular microenvironment autoimmunity contributes to the pathogenesis of POI. Follicular fluid contains a variety of autocrine and paracrine factors responsible for the regulation of folliculogenesis, oocyte development, and ovarian function.^8^, ^9^, ^10^, ^11^ Resident and infiltrating leukocytes are the principal sources of cytokine synthesis into FF and surrounding tissue. Additionally, ovarian somatic cells, including luteal, stromal, thecal, and granulosa cells, are an important cellular source as well.^11^ The abundance or dysregulation may play a role in ovarian insufficiency. The marked difference specific in FF profile between control and bPOI is striking, suggesting a distinct progression status of ovarian insufficiency in bPOI. Although we cannot accurately describe the contribution of each cytokine, the abnormally elevated chemokine levels in the FF may adversely impact oocyte quality and GC/theca cell function by cellular and paracrine interactions and inducing intracellular inflammatory processes in the follicular niche. They could also attract massive influx of leukocytes or induce greater immune cell activation, thus contributing to a chronic low‐grade inflammatory state, which in turn aggravates the follicle apoptosis and atresia and eventually affect the quality and quantity of oocyte.

Chemokines, or chemotactic cytokines, are crucial to the homeostasis of immunity by chemotaxis of responsive cells, especially the leukocytes.^16^ In the FF of patients with bPOI, MIP‐1α, CXCL8, IP‐10, and eotaxin‐1 were significantly elevated. Chemokines IP‐10, MIP‐1α, and IL‐8 mainly recruited NK cell, macrophage, and neutrophil influx, respectively, and have been suggested to be responsible for mediated and enhanced Th1 immune response.^16^, ^17^ They were considered as potent neutrophil chemotactic, and activating factors have been suggested to be one of the most likely agents responsible for the recruitment and activation of neutrophils in and around the pre‐ovulatory follicle just before ovulation. As important pro‐inflammatory mediators, they have been reported in association with many inflammatory diseases, such as endometriosis, autoimmune thyroiditis, systemic lupus erythematosus, psoriasis, and rheumatoid arthritis.^18^, ^19^, ^20^ The presence of autoimmune oophoritis was found in POI patients and mice models, with characteristic recruitment and infiltration of T lymphocytes and macrophages, plasma neutrophils, and NK cells. The accumulation of inflammatory chemokines might attract infiltration and induce activation of leukocytes in ovary, resulting in subsequent progressive decrease of ovarian function and reserve. eotaxin‐1 mainly engaged in eosinophil and basophil migration.^16^ eotaxin‐1/CCR3 axis was transiently upregulated in the ovarian theca‐interstitial layer in response to LH surge during the terminal stages of follicular development.^21^ It induced oxidative stress‐related tissue damage by promoting ROS production in a dose‐dependent manner by eosinophil activation.^22^, ^23^, ^24^ Moreover, eotaxin‐1 was increased in plasma and cerebral spinal fluid of healthy aging human and correlated with reduced neurogenesis in aged mice.^25^ It could also directly impair Schwann cell remyelination.^26^ Therefore, eotaxin‐1 might be an age‐related factor and contributor for the aging process, including premature aging of oocyte, even except for its chemotactic function. Further researches are warranted to confirm the association and explore the possible mechanism.

Chemokine RANTES played a critical role in attracting macrophage and NK cell migration and intermediating interaction between T cell and dendritic cell.^16^ It had been found in human follicular fluid and could be produced by granulosa cells and infiltrated leukocytes.^27^ Different from other chemokines, RANTES was identified significantly decreased in patients with bPOI. Interestingly, the level of RANTES was positively correlated with the rate of day 3 good‐quality embryo, which implied the reduced RANTES was associated with impaired quality of oocyte and embryo. In contrast, Shen et al^28^ found that excessive RANTES secreted by senescent theca‐interstitial cells might promote granulosa cell apoptosis and thus suppress follicular development and oocyte maturation. Yet they only consisted data from animal experiments but lacked clinical evidence. It still needs confirmation in other cohorts and more exploration for the underling mechanism.

In addition, we found growth factors BDNF, LIF, VEGF‐D and bFGF were significantly increased in follicular fluid in women with bPOI. BDNF, a member of the neurotrophin growth factors family, could promote oocyte maturation and embryo development and was also associated with infertility and endometriosis.^29^, ^30^, ^31^, ^32^ Czyzyk et al^33^ found lower plasma BDNF level in overt POI patients (FSH > 40 IU/L). Different stage of disease, sample source, and small sample size might explain the data inconsistence. LIF was reported to promote transition of primordial follicle to primary follicle, growth of pre‐antral follicles as well as oocyte maturation.^34^, ^35^, ^36^, ^37^ bFGF and VEGF‐D were involved in angiogenesis and follicle development in ovary.^38^, ^39^, ^40^, ^41^, ^42^ The increase of growth factors in bPOI might be associated with inflammatory tissue repair or compensatory response of growth activation in follicular microenvironment. At early stage of decline of ovarian function, there was a compensatory elevation of pro‐growth factors to promote follicle growth, oocyte maturation, and ovarian angiogenesis, whereas when ovarian reserve was completely exhausted, those factors would decrease along with estrogen.^43^, ^44^

As for cytokines, they preferentially showed significant difference in serum in patients with bPOI. Previous studies found increased inflammatory cytokines IL‐6, IFN‐γ, TNF‐α in human POI patients or POI mouse models.^45^, ^46^, ^47^ In our study, elevated level of IP‐10, IL‐7, IL‐17A but decreased IL‐1α and IL‐21 in peripheral suggested that systemic immune dysregulation might also exist in patients with bPOI. Unexpectedly, the typical pro‐inflammatory or immune regulatory cytokines turned out to exhibit no statistical differences in follicular fluid. What's more, IP‐10 was found increased in FF and paired serum in patients with bPOI, both of which showed significant association with biomarkers of ovarian reserve. These data suggested that chemokine IP‐10 could be considered as a potential autoimmune indicator for patients with bPOI. Given lack of strong correlation for most cytokines in FF and paired serum, as previously reported in a recent PCOS study,^48^ circulatory level was not equivalent to local inflammatory environment in the ovarian follicles. The local inflammatory milieu may play more important roles for oocyte quality and ovarian function. Different from the classical autoimmune disease, bPOI might be more inclined to immune disturbance of local ovarian microenvironment, as indicated by preferential dysregulation of chemokine and growth factors here. Future studies should focus more on the local ovarian environment for autoimmune pathogenesis of POI.

Previous studies, with quite smaller sample size, only focused on overt POI patients with FSH > 40 IU/L but not bPOI patients at earlier stage. Moreover, those studies detected cytokines in sera but seldom involving cytokines in follicular fluids.^33^, ^45^ In addition, it is generally not the presence or absence of any one cytokine that is responsible for the resultant immune response, rather the collective effect of the “cytokine milieu,” or cytokine microenvironment surrounding the effector cell. To our knowledge, it was the very first study that multiple inflammatory cytokines, chemokines, and growth factors were simultaneously detected and a comprehensive picture of the cytokine profile in follicular fluid and paired serum in bPOI was drawn. We emphasized the role cytokines especially chemokines might play in the pathophysiology of POI, thus opened a new field of exploration related to autoimmunity for clinician and scientists to pay attention to. There were a few limitations in this study. Firstly, the sample size was relatively small, which led to inadequate statistical power. The heterogeneity of individuals might also obscure the trends. Secondly, several cytokines showed very low level especially in follicular fluid, which made results less reliable and more difficult to be statistically significant between two groups. This partially might due to the multiplex immunoassay with magnetic beads used.

Taken together, altered cytokine profile characterized by increased chemokines and growth factors was associated with early stage of POI, which may fuel the progression of this disease or even play a crucial and direct role in the development of ovarian insufficiency. These data provide more evidence supporting the role of autoimmunity in the etiology of POI and promise a possible effect of immuno‐modulating therapy for ovarian resumption in a certain group of POI patients. Further exploration of clinical trials in larger sample size and basic studies for underling mechanisms are still needed.

## CONFLICT OF INTEREST

The authors report no conflict of interest.

## Supporting information

Supplementary Material
