# Supplementary material for: Dysregulated cytokine profile associated with biochemical premature ovarian insufficiency
Source: Am J Reprod Immunol. 2020 Jun 26;84(4):e13292. doi: 10.1111/aji.13292 (PMC7539985; doi:10.1111/aji.13292)
Supplement: Supplementary file 1 — Supplementary Material [file AJI-84-e13292-s001.docx]

**Supplementary**

**Supplementary Table 1. Cytokine profile in follicular fluid between patients with bPOI and control women.**

| Follicular fluid variables (pg/ml) | bPOI | CON | P value |
| --- | --- | --- | --- |
| MIP-1α | 1.15 (0.78-2.29) | 0.91 (0.41-1.60) | 0.043* |
| SDF-1α | 68.31 (32.17-86.13) | 61.84 (39.80-104.59) | 0.398 |
| IL-27 | 10.02 (4.29-16.27) | 10.02 (4.29-10.02) | 0.020* |
| LIF | 74.77 (50.53-153.09) | 56.85 (33.42-80.41) | 0.002* |
| IL-1β | 1.01 (1.01-1.65) | 1.01 (1.01-1.01) | 0.059 |
| IL-2 | 2.77 (2.77-5.35) | 2.77 (2.77-8.83) | 0.453 |
| IL-4 | 4.86 (1.75-7.89) | 1.75 (1.75-4.86) | 0.097 |
| IL-5 | 0.47 (0.47-0.77) | 0.47 (0.47-0.77) | 0.847 |
| IP-10 | 14.79 (11.01-21.39) | 13.09 (8.81-18.89) | 0.041* |
| IL-6 | 5.70 (5.70-8.31) | 5.70 (5.70-8.31) | 0.691 |
| IL-7 | 0.32 (0.20-1.03) | 0.26 (0.09-0.54) | 0.105 |
| CXCL8 | 5.76 (4.53-8.30) | 4.46 (2.97-6.37) | 0.024* |
| IL-10 | 0.06 (0.06-0.12) | 0.06 (0.03-0.10) | 0.155 |
| PIGF-1 | 2.43 (1.42-5.19) | 1.99 (0.92-4.75) | 0.193 |
| Eotaxin-1 | 7.07 (3.62-39.67) | 5.12 (2.18-12.40) | 0.015* |
| IL-12p70 | 0.11 (0.11-0.20) | 0.11 (0.03-0.18) | 0.513 |
| IL-13 | 3.83 (2.30-3.83) | 3.83 (2.30-3.83) | 0.547 |
| IL-17A | 0.71 (0.17-0.71) | 0.71 (0.17-0.71) | 0.295 |
| IL-31 | 2.15 (1.32-2.97) | 2.15 (1.32-2.15) | 0.558 |
| IL-1RA | 199.13 (176.70-221.74) | 199.13 (176.70-244.51) | 0.544 |
| SCF | 1.73 (1.23-2.52) | 1.73 (0.98-2.34) | 0.244 |
| RANTES | 3.31 (1.40-8.24) | 6.26 (4.22-8.84) | 0.006* |
| IFN-γ | 0.63 (0.20-1.51) | 0.63 (0.63-1.07) | 0.402 |
| GM-CSF | 9.81 (9.81-9.81) | 9.81 (4.96-9.81) | 0.879 |
| TNF-α | 1.24 (0.77-1.63) | 1.24 (0.86-2.18) | 0.479 |
| HGF | 813.46 (644.40-957.60) | 694.61 (633.21-867.47) | 0.190 |
| MIP-1β | 8.37 (6.61-15.47) | 8.88 (6.71-12.40) | 0.981 |
| IFN-α | 0.03 (0.02-0.03) | 0.02 (0.02-0.04) | 0.610 |
| MCP-1 | 23.42 (21.93-28.49) | 23.42 (21.93-24.87) | 0.146 |
| IL-9 | 1.77 (0.98-1.77) | 1.77 (0.98-1.77) | 0.845 |
| VEGF-D | 1.95 (1.64-2.29) | 1.68 (1.25-2.22) | 0.047* |
| TNF-β | 0.72 (0.43-0.72) | 0.72 (0.43-0.72) | 0.255 |
| bNGF | 8.01 (6.01-9.68) | 6.01 (6.01-8.01) | 0.086 |
| EGF | 2.04 (1.41-2.04) | 1.41 (0.78-2.04) | 0.054 |
| BDNF | 16.26 (7.98-37.93) | 8.79 (4.56-21.91) | 0.043* |
| GRO-α | 8.19 (2.40-13.71) | 7.21 (4.44-12.96) | 0.406 |
| IL-1α | 0.16 (0.12-0.26) | 0.15 (0.08-0.24) | 0.209 |
| IL-23 | 1.23 (1.23-2.12) | 1.23 (0.81-1.23) | 0.297 |
| IL-15 | 2.00 (0.05-3.69) | 2.00 (0.53-2.00) | 0.844 |
| IL-18 | 2.77 (2.20-3.33) | 2.77 (2.20-2.77) | 0.995 |
| IL-21 | 2.77 (1.49-6.47) | 2.77 (1.72-6.18) | 0.713 |
| bFGF | 11.45 (8.20-15.56) | 9.16 (6.05-11.45) | 0.046* |
| IL-22 | 47.79 (24.04-73.29) | 47.79 (47.79-73.29) | 0.085 |
| PDGF-BB | 3.99 (3.99-6.47) | 3.99 (3.99-6.47) | 0.589 |
| VEGF-A | 1734.38 (776.03-2434.48) | 1538.27 (841.55-1923.57) | 0.121 |

BDNF, brain-derived neutrophic factor; Eotaxin-1/CCL11; bFGF, basic fibroblast growth factor; EGF, epidermal growth factor; GM-CSF, granulocyte-macrophage colony-stimulating factor; GRO, growth related oncogene; HGF, hepatocyte growth factor; bNGF, nerve Growth Factor-β; LIF, Leukemia inhibitory factor; IFN, interferon; IP, interferon inducible protein; MCP, monocyte chemotactic protein; MIP, macrophage inflammatory protein; SDF, stromal cell derived factor; TNF, tumor necrosis factor; PDGF, platelet-derived growth factor; PIGF, placenta growth factor; SCF, stem cell factor; VEGF, vascular endothelial growth factor; IL, interleukin.

**Supplementary Table 2. Cytokine profile in serum between patients with bPOI and control women.**

| Serum variables (pg/ml) | bPOI | CON | P value |
| --- | --- | --- | --- |
| MIP-1α | 5.07 (2.86-20.46) | 4.24 (2.35-7.34) | 0.184 |
| SDF-1α | 289.27 (252.90-342.55) | 307.16 (249.90-394.34) | 0.589 |
| IL-27 | 198.36 (156.89-198.36) | 198.36 (156.89-198.36) | 0.806 |
| LIF | 4.06 (4.06-5.44) | 4.06 (3.70-6.76) | 0.805 |
| IL-1β | 3.79 (3.79-5.62) | 3.79 (3.79-3.79) | 0.640 |
| IL-2 | 7.31 (7.31-7.31) | 7.31 (0.31-7.31) | 0.364 |
| IL-4 | 78.18 (52.58-78.18) | 78.18 (52.58-78.18) | 0.843 |
| IL-5 | 0.90 (0.90-1.68) | 0.90 (0.19-0.90) | 0.285 |
| IP-10 | 245.97 (188.38-352.99) | 210.95 (157.06-262.35) | 0.032* |
| IL-6 | 15.99 (8.85-15.99) | 15.99 (8.85-15.99) | 0.779 |
| IL-7 | 5.12 (2.93-6.96) | 3.75 (2.34-5.12) | 0.027* |
| CXCL8 | 6.65 (3.21-11.91) | 5.37 (3.21-9.03) | 0.440 |
| IL-10 | 0.32 (0.20-0.32) | 0.20 (0.20-0.32) | 0.056 |
| PIGF-1 | 52.51 (39.99-66.09) | 58.83 (39.48-85.23) | 0.414 |
| Eotaxin-1 | 6.04 (4.30-9.70) | 5.64 (3.23-8.22) | 0.329 |
| IL-12p70 | 0.40 (0.40-0.86) | 0.40 (0.40-0.57) | 0.821 |
| IL-13 | 9.98 (5.50-9.98) | 9.98 (5.50-9.98) | 0.800 |
| IL-17A | 8.36 (5.35-11.34) | 5.35 (5.35-7.60) | 0.017* |
| IL-31 | 8.60 (6.69-10.51) | 8.60 (6.69-10.51) | 0.954 |
| IL-1RA | 814.88 (814.88-814.88) | 814.88 (729.92-814.88) | 0.667 |
| SCF | 1.49 (0.93-2.07) | 1.49 (0.93-2.95) | 0.228 |
| RANTES | 207.11 (179.42-249.12) | 207.90 (172.40-251.79) | 0.768 |
| IFN-γ | 12.32 (9.77-14.78) | 9.77 (9.77-14.78) | 0.766 |
| GM-CSF | 22.68 (16.44-28.36) | 16.44 (16.44-28.36) | 0.688 |
| TNF-α | 1.94 (1.94-2.07) | 1.94 (1.94-2.97) | 0.600 |
| HGF | 51.41 (34.84-70.99) | 43.92 (24.50-73.23) | 0.574 |
| MIP-1β | 38.29 (14.76-118.72) | 43.63 (24.83-80.69) | 0.376 |
| IFN-α | 0.07 (0.04-0.07) | 0.04 (0.04-0.07) | 0.830 |
| MCP-1 | 16.20 (16.20-22.00) | 19.12 (16.20-24.14) | 0.254 |
| IL-9 | 12.44 (9.42-13.21) | 12.44 (9.42-15.53) | 0.981 |
| VEGF-D | 1.26 (1.26-2.06) | 1.26 (1.26-2.87) | 0.161 |
| TNF-β | 0.30 (0.10-0.55) | 0.30 (0.30-0.55) | 0.166 |
| bNGF | 10.83 (4.30-10.83) | 10.83 (4.30-18.51) | 0.580 |
| EGF | 6.03 (3.61-10.69) | 3.61 (6.03-8.39) | 0.797 |
| BDNF | 171.80 (106.85-256.47) | 173.82 (104.80-273.70) | 0.974 |
| GRO-α | 19.83 (13.45-26.92) | 21.31 (11.72-29.22) | 0.854 |
| IL-1α | 0.46 (0.28-0.62) | 0.66 (0.35-0.89) | 0.044* |
| IL-23 | 11.47 (10.55-11.47) | 11.47 (4.01-11.47) | 0.444 |
| IL-15 | 4.42 (4.42-6.83) | 4.42 (4.42-6.83) | 0.821 |
| IL-18 | 8.90 (5.46-12.11) | 8.90 (5.46-11.71) | 0.625 |
| IL-21 | 23.54 (9.75-47.05) | 42.70 (17.42-68.97) | 0.042* |
| bFGF | 43.02 (37.06-55.10) | 49.04 (40.04-55.10) | 0.390 |
| IL-22 | 80.38 (20.11-80.38) | 80.38 (20.11-80.38) | 0.861 |
| PDGF-BB | 17.30 (13.38-23.22) | 18.29 (13.38-25.20) | 0.641 |
| VEGF-A | 63.16 (47.07-121.67) | 74.70 (46.42-124.22) | 0.465 |

BDNF, brain-derived neutrophic factor; Eotaxin-1/CCL11; bFGF, basic fibroblast growth factor; EGF, epidermal growth factor; GM-CSF, granulocyte-macrophage colony-stimulating factor; GRO, growth related oncogene; HGF, hepatocyte growth factor; bNGF, nerve Growth Factor-β; LIF, Leukemia inhibitory factor; IFN, interferon; IP, interferon inducible protein; MCP, monocyte chemotactic protein; MIP, macrophage inflammatory protein; SDF, stromal cell derived factor; TNF, tumor necrosis factor; PDGF, platelet-derived growth factor; PIGF, placenta growth factor; SCF, stem cell factor; VEGF, vascular endothelial growth factor; IL, interleukin.

**Supplementary Table 3. Correlations between follicular fluid cytokines and biomarkers of ovarian reserve**

| Follicular fluid cytokines (pg/ml) | FSH (IU/L) | | AMH (ng/ml) | | AFC | |
| --- | --- | --- | --- | --- | --- | --- |
|  | Spearman's rho | P value | Spearman's rho | P value | Spearman's rho | P value |
| MIP-1α | 0.302 | 0.012* | -0.353 | 0.004* | -0.278 | 0.021* |
| SDF-1α | -0.075 | 0.537 | -0.034 | 0.786 | 0.037 | 0.759 |
| IL-27 | 0.278 | 0.018* | -0.329 | 0.006* | -0.328 | 0.005* |
| LIF | 0.367 | 0.002* | -0.325 | 0.007* | -0.309 | 0.008* |
| IL-1β | 0.195 | 0.100 | -0.236 | 0.051 | -0.225 | 0.057 |
| IL-2 | -0.102 | 0.395 | 0.093 | 0.446 | -0.103 | 0.388 |
| IL-4 | 0.199 | 0.099 | -0.282 | 0.021* | -0.177 | 0.142 |
| IL-5 | 0.011 | 0.925 | -0.084 | 0.498 | -0.020 | 0.867 |
| IP-10 | 0.254 | 0.033* | -0.118 | 0.337 | -0.211 | 0.077 |
| IL-6 | 0.104 | 0.390 | -0.169 | 0.169 | -0.223 | 0.062 |
| IL-7 | 0.158 | 0.187 | -0.121 | 0.324 | -0.108 | 0.372 |
| CXCL8 | 0.202 | 0.090 | -0.305 | 0.011* | -0.297 | 0.012* |
| IL-10 | 0.278 | 0.021* | -0.233 | 0.059 | -0.241 | 0.046* |
| PIGF-1 | 0.233 | 0.050 | -0.311 | 0.010* | -0.356 | 0.002* |
| Eotaxin-1 | 0.334 | 0.004* | -0.392 | 0.001* | -0.323 | 0.006* |
| IL-12p70 | 0.109 | 0.362 | -0.188 | 0.121 | -0.131 | 0.274 |
| IL-13 | 0.175 | 0.140 | -0.209 | 0.085 | -0.228 | 0.054 |
| IL-17A | 0.154 | 0.204 | -0.161 | 0.194 | -0.067 | 0.579 |
| IL-31 | 0.195 | 0.103 | -0.237 | 0.052 | -0.239 | 0.044* |
| IL-1RA | -0.091 | 0.449 | 0.021 | 0.867 | -0.041 | 0.737 |
| SCF | 0.144 | 0.231 | -0.146 | 0.233 | -0.279 | 0.018* |
| RANTES | -0.324 | 0.005* | 0.349 | 0.003* | 0.288 | 0.014* |
| IFN-γ | 0.090 | 0.461 | -0.311 | 0.011* | -0.078 | 0.522 |
| GM-CSF | -0.009 | 0.939 | -0.053 | 0.672 | -0.026 | 0.831 |
| TNF-α | -0.108 | 0.372 | 0.034 | 0.782 | 0.084 | 0.487 |
| HGF | 0.285 | 0.018* | -0.196 | 0.114 | -0.330 | 0.006* |
| MIP-1β | 0.060 | 0.625 | -0.134 | 0.278 | -0.058 | 0.634 |
| IFN-α | 0.134 | 0.268 | -0.142 | 0.253 | -0.247 | 0.040* |
| MCP-1 | 0.193 | 0.110 | -0.230 | 0.061 | -0.213 | 0.076 |
| IL-9 | 0.107 | 0.377 | -0.132 | 0.284 | -0.248 | 0.037* |
| VEGF-D | 0.305 | 0.010* | -0.258 | 0.033* | -0.344 | 0.003* |
| TNF-β | 0.146 | 0.224 | -0.231 | 0.058 | -0.285 | 0.016* |
| bNGF | 0.295 | 0.013* | -0.289 | 0.017* | -0.274 | 0.021* |
| EGF | 0.257 | 0.030* | -0.352 | 0.003* | -0.218 | 0.068* |
| BDNF | 0.325 | 0.006* | -0.323 | 0.008* | -0.342 | 0.004* |
| GRO-α | 0.085 | 0.482 | -0.074 | 0.550 | -0.052 | 0.668 |
| IL-1α | 0.233 | 0.051 | -0.281 | 0.020* | -0.260 | 0.028* |
| IL-23 | 0.156 | 0.189 | -0.192 | 0.115 | -0.184 | 0.122 |
| IL-15 | 0.082 | 0.498 | -0.169 | 0.171 | -0.227 | 0.059 |
| IL-18 | 0.049 | 0.683 | -0.156 | 0.201 | -0.154 | 0.197 |
| IL-21 | 0.119 | 0.328 | -0.216 | 0.079 | -0.268 | 0.026* |
| bFGF | 0.253 | 0.036* | -0.320 | 0.009* | -0.292 | 0.015* |
| IL-22 | -0.200 | 0.091 | 0.213 | 0.079 | 0.195 | 0.100 |
| PDGF-BB | -0.047 | 0.695 | 0.008 | 0.950 | 0.031 | 0.797 |
| VEGF-A | 0.236 | 0.046* | -0.177 | 0.146 | -0.291 | 0.013* |

BDNF, brain-derived neutrophic factor; Eotaxin-1/CCL11; bFGF, basic fibroblast growth factor; EGF, epidermal growth factor; GM-CSF, granulocyte-macrophage colony-stimulating factor; GRO, growth related oncogene; HGF, hepatocyte growth factor; bNGF, nerve Growth Factor-β; LIF, Leukemia inhibitory factor; IFN, interferon; IP, interferon inducible protein; MCP, monocyte chemotactic protein; MIP, macrophage inflammatory protein; SDF, stromal cell derived factor; TNF, tumor necrosis factor; PDGF, platelet-derived growth factor; PIGF, placenta growth factor; SCF, stem cell factor; VEGF, vascular endothelial growth factor; IL, interleukin.

**Supplementary Table 4. Correlations between serum cytokines and biomarkers of ovarian reserve**

| Serum  cytokines (pg/ml) | FSH (IU/L) |  | AMH (ng/ml) |  | AFC |  |
| --- | --- | --- | --- | --- | --- | --- |
|  | Spearman's rho | P value | Spearman's rho | P value | Spearman's rho | P value |
| MIP-1α | 0.225 | 0.062 | -0.077 | 0.534 | -0.197 | 0.102 |
| SDF-1α | 0.003 | 0.981 | -0.046 | 0.710 | -0.128 | 0.284 |
| IL-27 | 0.025 | 0.837 | -0.143 | 0.244 | -0.008 | 0.947 |
| LIF | -0.025 | 0.836 | 0.030 | 0.804 | -0.038 | 0.753 |
| IL-1β | 0.500 | 0.684 | -0.095 | 0.446 | -0.130 | 0.289 |
| IL-2 | 0.057 | 0.631 | -0.142 | 0.243 | -0.112 | 0.350 |
| IL-4 | 0.039 | 0.744 | 0.000 | 0.997 | -0.099 | 0.410 |
| IL-5 | 0.143 | 0.234 | -0.135 | 0.271 | -0.032 | 0.790 |
| IP-10 | 0.284 | 0.017* | -0.216 | 0.079 | -0.263 | 0.028* |
| IL-6 | 0.024 | 0.841 | -0.161 | 0.191 | -0.141 | 0.240 |
| IL-7 | 0.180 | 0.141 | -0.172 | 0.172 | -0.154 | 0.211 |
| CXCL8 | 0.103 | 0.395 | 0.020 | 0.873 | -0.278 | 0.020* |
| IL-10 | 0.227 | 0.059 | -0.274 | 0.025* | -0.188 | 0.119 |
| PIGF-1 | -0.056 | 0.645 | 0.039 | 0.751 | 0.066 | 0.586 |
| Eotaxin-1 | 0.169 | 0.162 | -0.169 | 0.170 | -0.191 | 0.114 |
| IL-12p70 | 0.111 | 0.357 | -0.200 | 0.102 | -0.107 | 0.372 |
| IL-13 | -0.028 | 0.817 | -0.082 | 0.503 | -0.014 | 0.908 |
| IL-17A | 0.302 | 0.010* | -0.350 | 0.003* | -0.192 | 0.109 |
| IL-31 | 0.021 | 0.858 | -0.019 | 0.876 | -0.063 | 0.598 |
| IL-1RA | 0.019 | 0.875 | -0.037 | 0.765 | -0.142 | 0.239 |
| SCF | -0.090 | 0.451 | 0.066 | 0.591 | -0.048 | 0.688 |
| RANTES | 0.058 | 0.626 | 0.023 | 0.851 | -0.155 | 0.192 |
| IFN-γ | -0.008 | 0.948 | -0.256 | 0.033* | -0.109 | 0.364 |
| GM-CSF | -0.028 | 0.818 | -0.024 | 0.846 | 0.048 | 0.686 |
| TNF-α | 0.013 | 0.914 | 0.052 | 0.673 | -0.046 | 0.702 |
| HGF | 0.000 | 0.998 | -0.054 | 0.662 | -0.098 | 0.418 |
| MIP-1β | -0.055 | 0.650 | 0.147 | 0.235 | -0.063 | 0.602 |
| IFN-α | -0.050 | 0.675 | -0.045 | 0.714 | 0.015 | 0.901 |
| MCP-1 | -0.144 | 0.235 | 0.032 | 0.798 | -0.032 | 0.790 |
| IL-9 | 0.097 | 0.420 | -0.051 | 0.681 | -0.129 | 0.282 |
| VEGF-D | -0.130 | 0.276 | 0.157 | 0.196 | 0.072 | 0.546 |
| TNF-β | -0.088 | 0.463 | 0.068 | 0.577 | 0.011 | 0.925 |
| bNGF | -0.096 | 0.431 | 0.102 | 0.413 | 0.056 | 0.647 |
| EGF | -0.054 | 0.656 | 0.019 | 0.878 | 0.048 | 0.694 |
| BDNF | 0.011 | 0.925 | -0.055 | 0.654 | -0.099 | 0.413 |
| GRO-α | -0.032 | 0.788 | -0.175 | 0.151 | -0.249 | 0.036* |
| IL-1α | -0.199 | 0.099 | 0.185 | 0.131 | 0.125 | 0.303 |
| IL-23 | 0.173 | 0.152 | -0.148 | 0.233 | -0.057 | 0.642 |
| IL-15 | 0.061 | 0.612 | -0.091 | 0.462 | -0.074 | 0.539 |
| IL-18 | 0.021 | 0.863 | -0.117 | 0.345 | -0.003 | 0.982 |
| IL-21 | -0.160 | 0.183 | 0.244 | 0.045* | 0.242 | 0.042* |
| bFGF | -0.077 | 0.523 | 0.095 | 0.443 | 0.106 | 0.379 |
| IL-22 | -0.036 | 0.765 | -0.154 | 0.206 | -0.092 | 0.440 |
| PDGF-BB | -0.095 | 0.433 | 0.109 | 0.382 | -0.031 | 0.798 |
| VEGF-A | -0.033 | 0.782 | 0.072 | 0.559 | 0.033 | 0.785 |

BDNF, brain-derived neutrophic factor; Eotaxin-1/CCL11; bFGF, basic fibroblast growth factor; EGF, epidermal growth factor; GM-CSF, granulocyte-macrophage colony-stimulating factor; GRO, growth related oncogene; HGF, hepatocyte growth factor; bNGF, nerve Growth Factor-β; LIF, Leukemia inhibitory factor; IFN, interferon; IP, interferon inducible protein; MCP, monocyte chemotactic protein; MIP, macrophage inflammatory protein; SDF, stromal cell derived factor; TNF, tumor necrosis factor; PDGF, platelet-derived growth factor; PIGF, placenta growth factor; SCF, stem cell factor; VEGF, vascular endothelial growth factor; IL, interleukin.

**
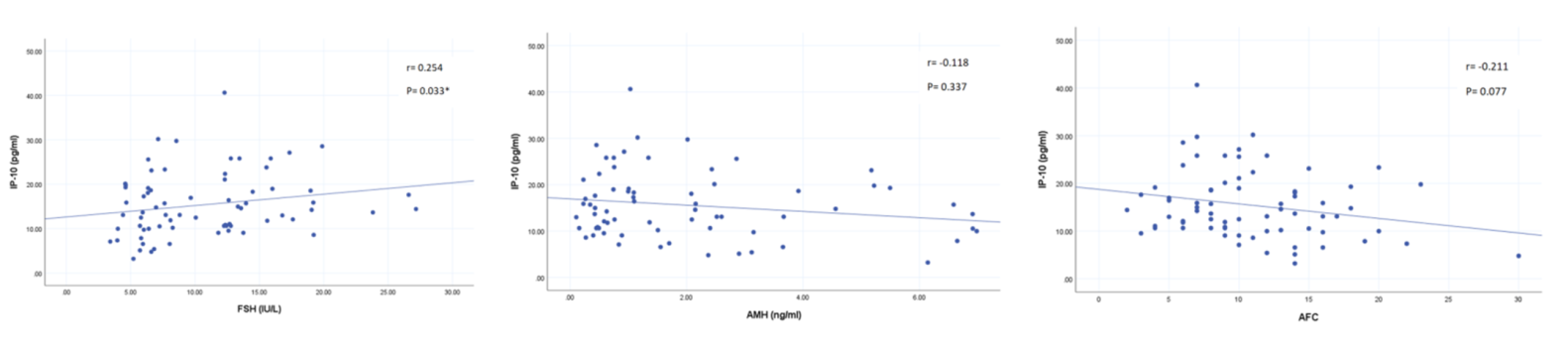

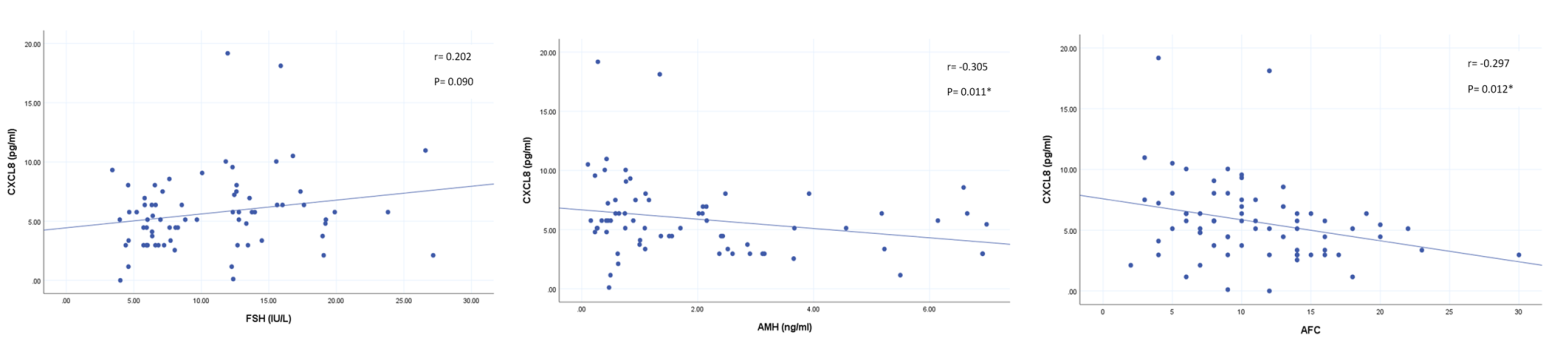

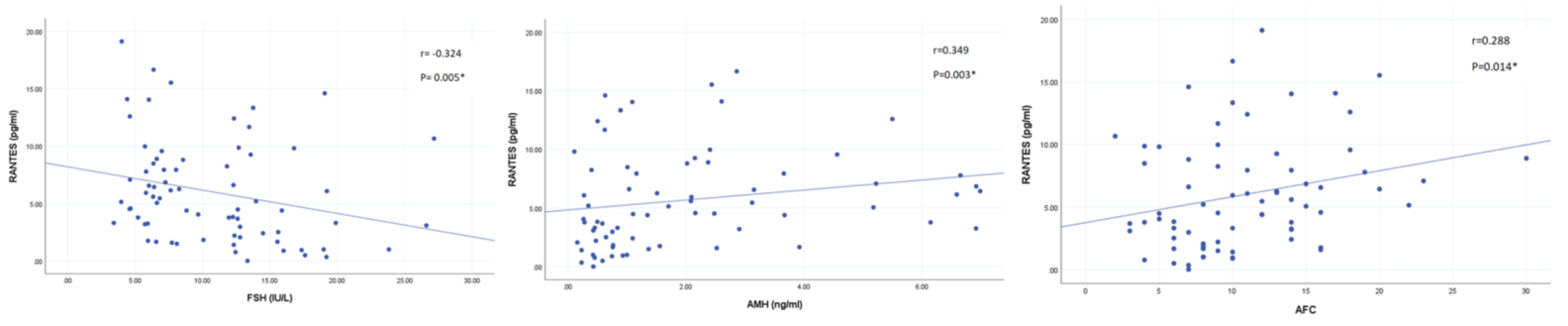

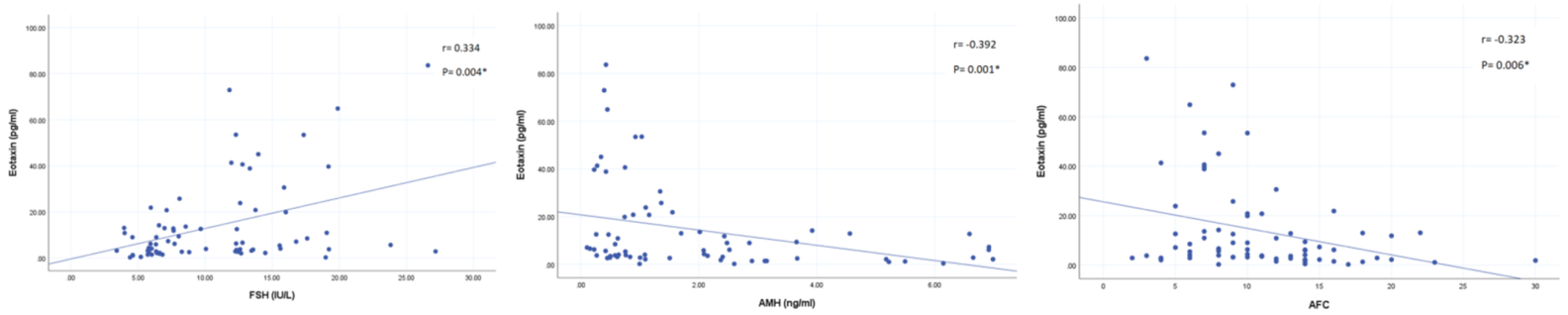

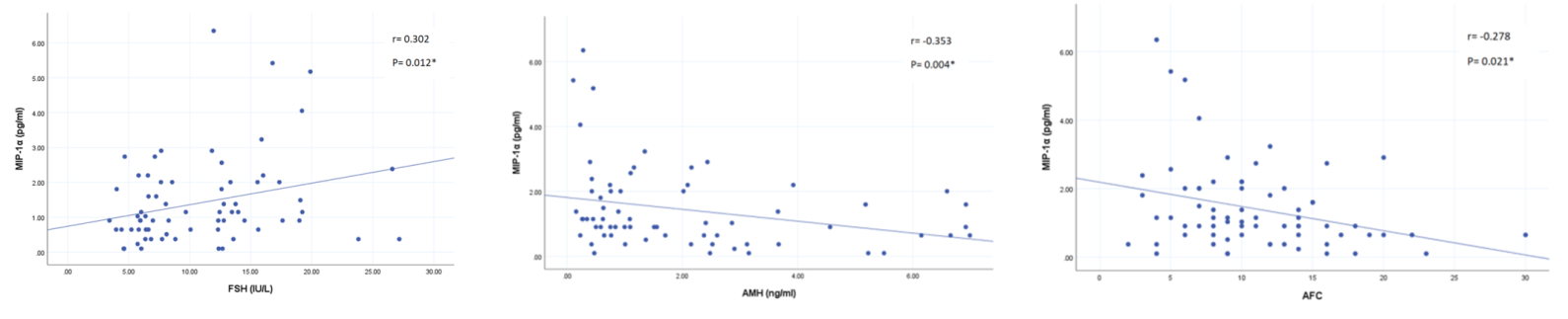
Supplementary Figure 1. Correlations between follicular fluid cytokines and biomarkers of ovarian reserve**

**
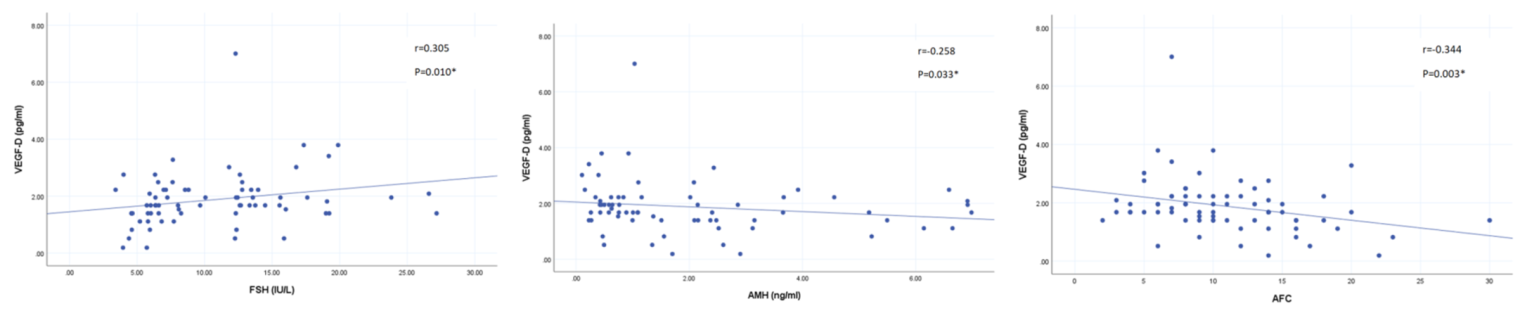

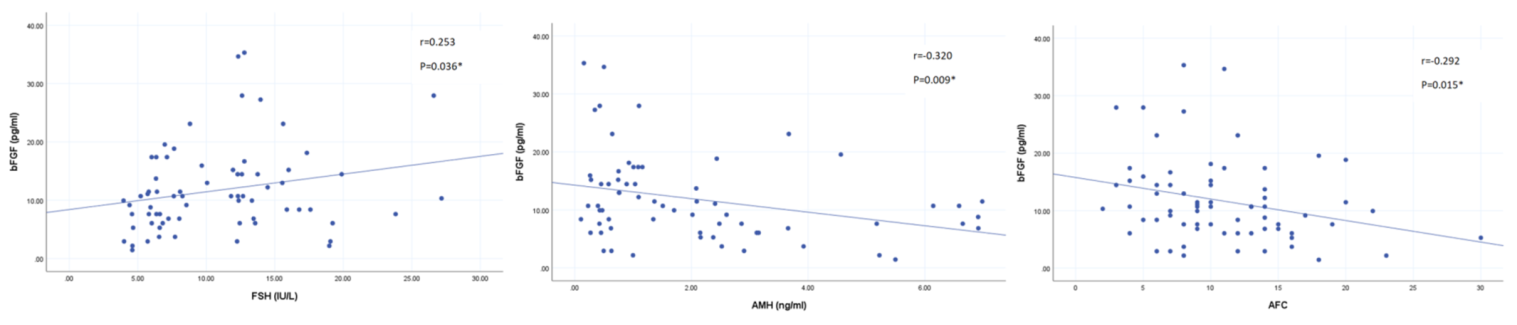

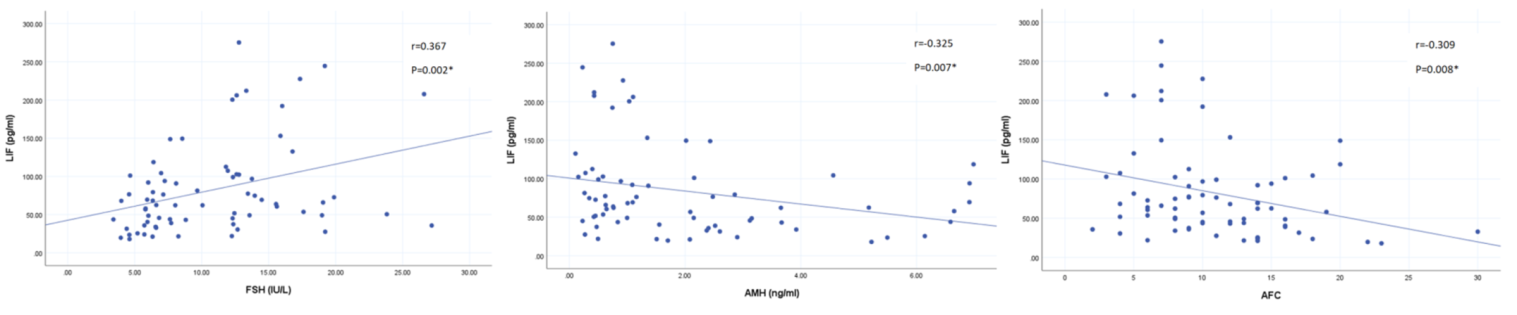

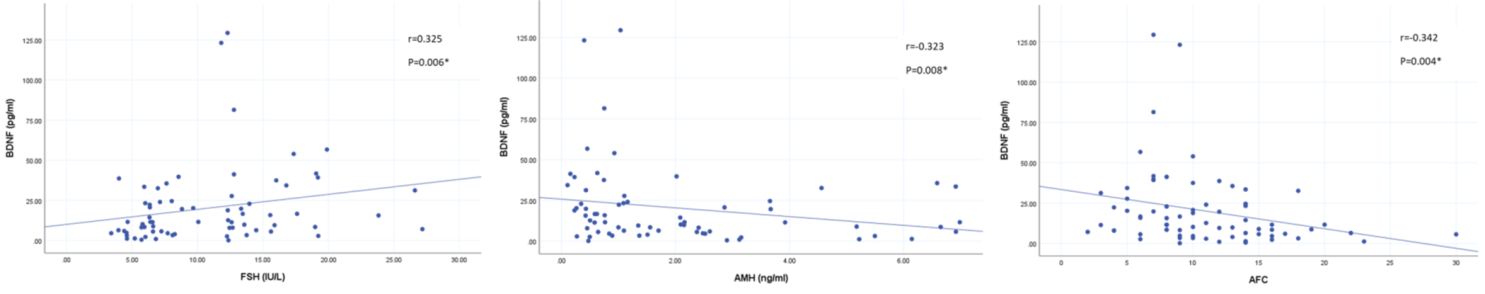
**MIP, macrophage inflammatory protein; IP, interferon inducible protein; BDNF, brain-derived neutrophic factor; LIF, Leukemia inhibitory factor; bFGF, basic fibroblast growth factor; VEGF, vascular endothelial growth factor.

Spearman’s correlation was used to estimate the association between cytokines in follicular fluid and the biomarkers of ovarian reserve.
